# Union in the Nursing Profession

**Published:** 1920-07-10

**Authors:** F. A. Sheldon

**Affiliations:** 14 St. Thomas's Street, S.E. 1


*To the Editor of* The Hospital.

### Union in the Nursing Profession.

*To the Editor of* The Hospital.

Sir,—Societies, the nursing Press, and individuals have joined in your protest against the action of the borough of Bermondsey in requiring their nurse employees to be members of a trade union. Most certainly political or religious tests are to be deprecated in connection with such appointments. The profession, however, awaits unification, and the registered nurse of the near future must realise that only by combination (call it by what name you will—College, Association, Union) can any standard of training, opportunity, and pay be raised and maintained. It is interesting to note that at the present moment a matron, a ward sister, and a district nurse, all personally known to me, are receiving from the Borough of Bermondsey a really adequate rate of pay and emoluments, enjoying longer holidays, and working shorter hours than have ever before been arranged for similar posts.—Yours faithfully, F. A. Sheldon.

14 St. Thomas's Street, S.E. 1,
July 5, 1920.

